# Evolution features of hypertensive patients with primary aldosteronism - Prospective Study

**Published:** 2012-09-25

**Authors:** V Chioncel, D Păun, B Amuzescu, C Sinescu

**Affiliations:** * „Bagdasar Arseni” Clinical Emergency Hospital, „Carol Davila” University of Medicine and Pharmacy, Bucharest; ** “C. I. Parhon” Endocrinology Institute, „Carol Davila” University of Medicine and Pharmacy, Bucharest; *** Biology Faculty, University of Bucharest

**Keywords:** secondary hypertension, primary aldosteronism, hypokalemia, surgery

## Abstract

**Background:** Primary aldosteronism is the leading cause of secondary hypertension, the management of this disease requiring an interdisciplinary approach.

**Objectives:** Evaluation of evolutionary features of patients with secondary hypertension and primary aldosteronism.

**Methodology:** We have followed 26 patients diagnosed with secondary hypertension and primary aldosteronism, who were admitted consecutively to "C. I. Parhon" Endocrinology Institute between 2004-2009. Of the 26 patients, 17 had adenoma producer of aldosterone (APA), 8 had bilateral adrenal hyperplasia idiopathic (HIA) and one patient had adrenal carcinoma (with hypersecretion of aldosterone). The mean age of the cohort was of 49.3 years (44.9 years for adenomas and 52.6 years for bilateral hyperplasia). The evaluation of the patients included clinical examination, electrocardiogram, Holter BP, echocardiography and determination of plasma aldosterone and renin.

**Results:** The evolution of the patients with primary aldosteronism was different depending on the anatomoclinic type. In patients with idiopathic bilateral hyperplasia, medical treatment has improved control of hypertension and cardiac and cerebrovascular complications rate was moderate. In patients with unilateral adenoma producing aldosterone, blood pressure had higher values and more frequent complications, but surgical cure of adenomas significantly changed the prognosis of patients. In both cases, the presence of hypokalemia was an additional element of severity.

**Conclusions:** Regardless of the primary aldosteronism, hypertension was directly involved in cardiac and cerebrovascular complications. Individualization of treatment according to the anatomoclinic type determined a significant improvement of the patients’ prognosis.

## Introduction

Described by Conn in 1956 [**1**], primary aldosteronism is the most common cause of secondary hypertension, current data suggest that this syndrome is responsible for over 10% of the cases of hypertension [**2,3**] and is present even in percent higher in patients with treatment-resistant hypertension. 

There are unilateral forms: 

- Aldosterone producing adenoma - APA - the most common form, 

- Unilateral adrenal hyperplasia 

- Adrenocortical carcinoma with secretion of aldosterone, and cases with bilateral adrenal location 

- Bilateral adrenal hyperplasia idiopathic - HIA 

- Glucocorticoid remediable aldosteronism (very rare form, when aldosterone secretion is induced by corticotropin and suppressed by glucocorticoids). 

With the highest prevalence, aldosterone-producing adenomas (APA) are those characterized by high values of blood pressure (which are resistant to usual therapy) and higher rate of complications. 

Although for a long time, it was considered a marker of suspicion almost mandatory for Conn syndrome, hypokalemia sensitivity as a diagnostic element is quite limited, current data revealing its presence in 9-37% of cases, more often in unilateral adenomas (~ 50%) [**4,5**].

The evolution of patients with primary aldosteronism is dictated by cardiac and cerebrovascular complications, as consequence of severity and lack of control of hypertension. Forms are very rare malignant (adrenocortical carcinoma) and are not characterized by a high rate of metastasis. 

If the diagnosis is established after clear several stages (biochemical tests revealed hypersecretion of aldosterone and hyperreninemia and imagistic evidence of adrenal formations), treatment should be nuanced depending on the location of unilateral or bilateral disease: surgery is the best choice in single adenomas, while for bilateral hyperplasia drug therapy is appropriate.

## Study objectives 

The progress of patients with secondary hypertension and primary aldosteronism based on anatomic and functional type and treatment. 

## Methodology

The study group included 26 patients with secondary hypertension who were diagnosed with primary aldosteronism. Patients were admitted consecutively between 2004-2009 at "C.I. Parhon" Institute of Endocrinology to establish the anatomo-clinic type of aldosteronism and therapeutic attitude.

Patients with surgical indication were operated at “Fundeni” Center of Urological Surgery and Renal Transplantation and "C. I. Parhon" Institute of Endocrinology. 

The study was of a prospective "case control" type, observational. Inclusion criteria were the presence of hypertension, biological determinations (increased levels of serum aldosterone and plasma renin low) and imaging (CT, MRI) suggestive for the diagnosis of primary aldosteronism.

Initial assessment protocol included clinical examination, ECG, Holter BP, echocardiography and measurements of aldosterone and plasma renin. Hormonal determinations were performed in laboratory of Fundeni Hospital, laboratory of "C. I. Parhon" Institute or 2 private laboratories (ISO certified). 

We used the method of radioimmunoassay (RIA) for the determination of plasma aldosterone, respectively immunochemical method with chemiluminescence detection for direct determination of plasma renin. 

Normal values of laboratory tests, which were made, are: 

 - Serum aldosterone: 

• supine (morning): 2.94 -15.1 ng / dL 

• standing: 3.81 - 31.3 ng / dL 

- Plasma renin: 

• supine: 0.168-2.39 ng / dL 

• standing: 0.26-2.76 ng / dL, 

with a normal value ratio aldosterone / plasma renin <20. 

Normal values of plasmatic K were 3,5-5,1 mEq/l. 

Measurements were made morning in supine, after stopping medication that could influence the levels of aldosterone and renin (diuretics, especially antialdosterone for 4 weeks and other antihypertensive like β-blockers, ACE inhibitors, α-central agonists for at least 2 weeks). 

We used the serum aldosterone report/plasma renin, with a cutoff of 45 as being highly suggestive of the diagnosis of primary aldosteronism [**6**]. 

The blood pressure measurement was done in supine, successively in both arms, after at least 5 minutes of rest, with approved blood pressure (cuff metrologically checked and appropriate in size), diagnosis of hypertension resulting from high values (over 140/90 mm Hg) at least two separate determinations.

Postoperatory patients were evaluated at 2 weeks, 6 months and 1 year by clinical examination and electrocardiography. At 2 weeks and 1 year after surgery, the hormone measurements were repeated; echocardiography was repeated at 6 months. 

Patients were followed for a period of between 8 months and 7 years, with an average of 3.8 years; there were 2 patients who did not return to control whose outcomes are unknown.

## Results

Of the 26 patients, 17 had aldosterone producing adenoma (APA), 8 had bilateral adrenal hyperplasia idiopathic (HIA) and a single patient was found with aldosterone adrenal carcinoma. 

From a demographic perspective, the average age in the study group was 49.3 years, range 32 to 68 years, lower in adenomas (44.9 years) and higher for bilateral hyperplasia (52,6 years); sex ratio was different depending on the type of primary aldosteronism: 70.6% women with unilateral adenoma, respectively 62.5% men for bilateral hyperplasia.

**Fig. 1:**
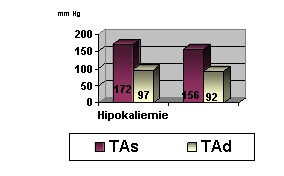
The relationship between hypertension and the serum potassium profile

In our group, hypokalemia was present in 15 of 26 patients (57.7%), more frequently in patients with adenomas with hypersecretion of aldosterone (13 of the 17 patients - 76.4%). Moreover, analyzing the profile of hypertensive patients, the presence of hypokalemia seems to correlate with higher values of blood pressure and thus a higher rate of cardiovascular complications (**Fig. 1**).

**Fig 2:**
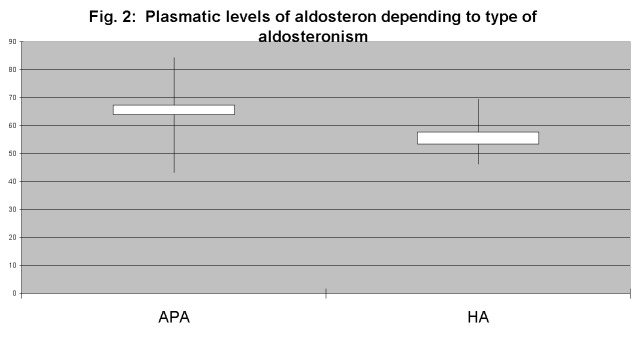


**Fig. 3:**
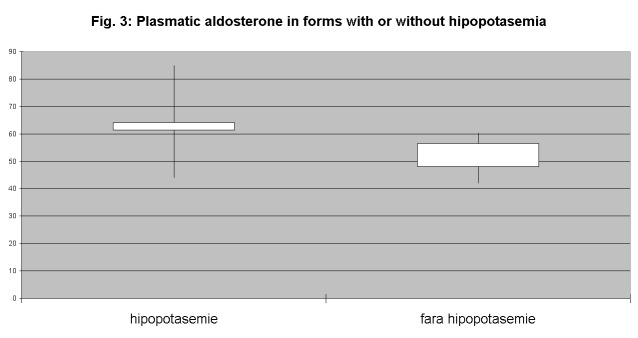


Analyzing aldosterone concentrations in the group of patients with aldosterone-producing adenomas, plasma levels have been consistently higher than at those with bilateral hyperplasia (**Fig. 2**). Also, the presence of hypokalemia was correlated with elevated serum concentrations of aldosterone (**Fig. 3**).

Patients with aldosterone-secreting adenomas had significantly higher blood pressure values than those with idiopathic bilateral hyperplasia: average values of systolic and diastolic blood pressure was 168+/-24 mm Hg and 95+/-13 mm Hg (at 24 hours Holter monitoring) versus 157+/- 26 mm Hg and 91+/-11 mm Hg in patients with bilateral hyperplasia, the mean systolic and diastolic for the entire group of patients with primary aldosteronism were 164+/-25 mm Hg, respectively 94+/-12 mm Hg. 

Until the diagnosis of primary aldosteronism, 24 of the 26 patients received antihypertensive treatment, which does not control blood pressure. 

Later (after clarifying diagnosis), all the 26 patients received antihypertensive treatment with drugs from several therapeutic classes (**Table 1**).

**Table 1:** Antihypertensive therapy in patients with Conn syndrome

CLASS OF ANTIHYPERTENSIVE	PERCENTAGE
ANTIALDOSTERONICS	100
ACE INHIBITORS	84,6%
Ca- BLOCKERS	73,1%
ANGIOTENSIN RECEPTOR BLOCKERS	61,5%
β-BLOCKERS	57,7%
α-BLOCKERS	46,2%

After elucidating the anatomo-functional type of aldosteronism, patients with unilateral tumors (17 with adenoma and one with adrenal carcinoma) were offered surgery and those with bilateral hyperplasia received medical treatment. 

Antihypertensive therapy: Normally all patients received therapy with a mineralocorticoid receptor blocker, most have received Spironolactone - 23 of 26 patients (mean dose 100 mg / day) and other 3 - Eplerenone (mean 125 mg / day ). Although without adverse effects of spironolactone (especially gynecomastia), reduced antialdosteronic effect and difficult accessibility (price and availability in pharmacies) have made a choice back for eplerenone. 

Patients received an average of 2.8 antihypertensive drugs, with differences between the two types of primary aldosteronism: adenoma hypersecretion (3.2) and bilateral adrenal hyperplasia [2,1]. 

There were differences between patients with and without hypokalemia, with the number of antihypertensive drugs: 3.1 for those with hypokalemia, respectively 2.4 drugs for others.

Patients with idiopathic bilateral hyperplasia were not candidates for surgery and they received further medical therapy. They were followed on average 3.6 years.

Goals of therapy were hypertension control and normalization of potassium and other electrolytes. 

The evolution of these patients and a patient with unilateral adenoma (who refused surgery) was relatively favorable, recorded one death (hemorrhagic stroke), 2 years after diagnosis. 

Control of hypertension was made with polytherapy, including an antialdosteronic drug and, often, an ACE inhibitor or angiotensin receptor blocker. 

On admission in the study, 7 of 9 patients with bilateral hyperplasia were uncontrolled hypertension (88.9%); after 1 year of treatment hypertension control rate was of 55.5% (5/9 patients). 

**Fig. 4:**
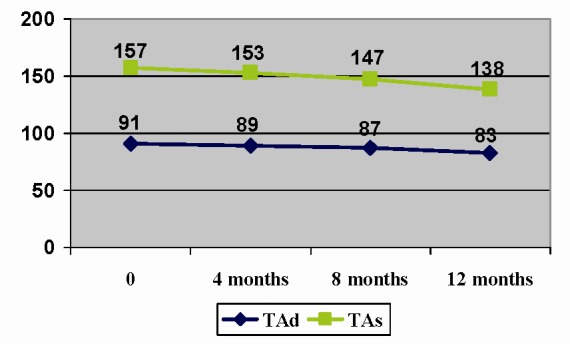
Evolution of hypertension in the first year of treatment

Also, lowering blood pressure in these patients was on average 19+/-11 mm Hg and 8+/-4 mm Hg (systolic value, diastolic respectively) after 1 year of treatment, maintaining the trend continued (**see Fig. 4**). 

The most notable complications of patients registered with idiopathic adrenal hyperplasia were:

 - Cardiovascular: 

- Severe hypertensive crisis (BP> 180/110 mm Hg) - 2 patients. 

- Acute cardiogenic pulmonary edema - 2 patients. 

- Arrhythmias - atrial fibrillation - one patient 

- Cerebrovascular: 

- Ischemic stroke - one patient 

- Hemorrhagic stroke - one patient, who died within 24 hours (at 2 years after diagnosis) 

 - Renal failure: 1 patient 

For patients with unilateral adenoma surgery was the best treatment option in most cases. 

17 patients were operated (16 with unilateral adenoma and one with adrenal carcinoma) after a mean period of 3.4 months, until then receiving antihypertensive therapy. 

Preoperative preparation of these 17 patients scheduled for intervention aimed decrease in BP and potassium level and consisted of administration - for 3-4 weeks - of Spironolactone (200-300 mg/day), associated with potassium supplements in patients with persistent hypokalemia. 

Surgery (performed on average 3.4 months from diagnosis) was the unilateral adrenalectomy, using laparoscopic technique (13 cases) or open laparotomy (4 cases). In all cases, the operation went smoothly, without significant complications and perioperative mortality was zero. 

Of postoperative complications, we noted hypertensive spikes (6 patients - 5 with APA and one with carcinoma) and postinterventional hypotension (4 cases with APA).

After surgery, hypoaldosteronism signs (hypokalemia, hyposodemia, metabolic acidosis) occurred in 5 cases, which do not require a special treatment, only adequate salt intake; these signs reversed in the first 3 months in 4 patients and after about 6 months in one patient. 

There were no distant metastases, but in the first year after surgery locoregional recurrence occurred in 2 cases (one carcinoma and one adenoma with hypersecretion), due most likely incomplete excision at baseline; reintervention was successful in both cases.

Efficiency of adrenalectomy was evident: in 9 patients (9/17 ~ 52.9%) resulting in cure of hypertension and in the other 8 cases easier to gain control of hypertension: one year after surgery the average number of antihypertensive drugs was 1.6 (from 3.2). 

**Fig. 5:**
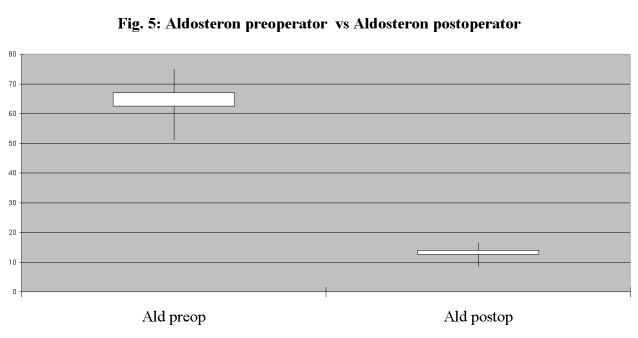


We also noted a significant decrease in plasma aldosterone at 1 year after surgery compared to preoperative values detected (**Fig. 5**): mean level of aldosterone was 12.44 ng/dL after surgery, compared with 62.7 ng/dL before adrenalectomy. 

All 8 patients who maintained hypertension (but with easier control after surgery) had hypokalemia; in contrast, only five of the nine patients who achieved cure of hypertension presented this dyselectrolytemia. 

There were three deaths during follow-up period (~ 11.5%) in the whole group of 26 patients with primary aldosteronism; mortality was similar for patients with unilateral adenomas (2/17 ~ 11.7%) and the bilateral hyperplasia (1/8 ~ 12.5%). Deaths have occurred through cardiac complications (myocardial infarction - 1 patient) or cerebrovascular (ischemic stroke - 1 patient and cerebral hemorrhage - 1 patient). 

All 3 patients who died (one operated) had hypokalemia, which is an additional argument to consider this mineral disorder as unfavorable prognostic factor in patients with primary aldosteronism.

## Discussions

Detection of primary aldosteronism is very important in clarifying the etiology of hypertension, especially because these patients have a higher rate of complications and a significant more severe evolution than patients with the same blood pressure values, but with essential hypertension [**7,8**]. 

 If 10 -15 years ago we believed that primary aldosteronism is a small fraction (<1%) of hypertensive patients, today many studies report the presence of Conn syndrome in over 10% of cases of hypertension [**9-14**]; also, hypokalaemia - once a mandatory marker - actually occurs in less than 50% of cases of primary aldosteronism [**4**]. 

 For positive diagnosis of primary aldosteronism must be met biochemical criteria (the relationship between serum aldosterone and plasma renin or plasma renin activity is the strongest evidence) and imagistic evidence (CT or MRI abdomen); in addition, other investigations (adrenal veins catheterization for determination of aldosterone level, adrenal postural test, NP59 scintigraphy with cholesterol) helps locate and determine the type of aldosteronism. 

 Hypertension and its complications marks the evolution of primary aldosteronism and that’s why hypertension is the main target of treatment. 

 The main weapon in the treatment of hypertension represents, as is natural, mineralocorticoid receptor antagonists, generally associated with other classes of antihypertensive agents (ACE inhibitors, angiotensin receptor blockers, Ca blockers, α-or β- blockers). 

 Beyond drug therapy in unilateral forms of primary aldosteronism, surgical excision of the tumor is the best option, contributing to the disappearance of hypertension or easier control of it.

 In our study, gender distribution was similar to the literature, adenomas appearing more often in women and bilateral hyperplasia more often in males. 

 Also, hypokalemia has not been a constant presence, appearing in 15 of 26 patients (57.7%); however, is a negative prognostic factor in the evolution of patients. 

 In our patients we have met more frequently adenomas producing aldosterone, but they are more severe than bilateral idiopathic hyperplasia: they appears in younger patients, with higher values of blood pressure, left ventricular hypertrophy and more frequently complications of hypertension.

 Evolution of patients with primary aldosteronism was directly related to high blood pressure profile and anatomic-functional type of aldosteronism. 

 In our patients, complications occurred in similar proportions like in other studies cited [**15**], authors of the German registry also noting a higher rate of cardiac or cerebral complications in patients with hypokalemia. 

 Treatment was individualized according to the type of hyperaldosteronism: 

 - Patients with unilateral formations (adenomas, carcinomas) received adrenalectomy, with good results: cure hypertension in 52% of cases and more easily control of blood pressure in the remaining patients. Although in the literature [**16,17**] surgical resolution of hypertension is described with a higher frequency (60-70%), lower percentage is probably due to greater age of hypertension in our patients. Resolution of hypertension after unilateral adrenectomy was obtained most frequently in patients without a family history of hypertension and those who used more than 2 antihypertensive medications before surgery; risk factors for persistent postoperative hypertension are: age over 45 years, high age (over 5 years) of hypertension and poor response to spironolactone [**18**]. Unilateral adrenalectomy was achieved predominantly laparoscopic (76.5% of cases) and there were no major complications or perioperative deaths. 

 - Those with bilateral idiopathic hyperplasia received medical treatment with mineralocorticoid receptor antagonists (associated with ACE inhibitors, angiotensin receptor blockers or Ca-blockers), with favorable outcome: after 1 year, 56% of them had good control of blood pressure, compared to 11% at baseline.

## Conclusions

Our study, although with a small number of patients, provides a useful data on this pathology. 

Although there is now clear that we must take into account Conn syndrome whenever investigating a new case of hypertension (especially in people under 45 years or abnormally high blood pressure, resistant to treatment), probably in Romania this pathology is still underdiagnosed. This is mainly due to the fact that hormonal determinations are not always readily available (price and availability), but also difficult to characterize further the type of primary aldosteronism (adrenal vein catheterization for hormonal dosage, genetic testing for identification of family forms, scintigraphy with NP59 cholesterol, etc.). 

In general, our findings overlap to those of other research from the last 10-15 years on this subject in terms of evolution data and details of treatment. 

The main limit of our research is the relatively small number of patients, thus not allowing complete statistical processing. 

Another limitation of the study is represented by incomplete exploration of patients, the main reason being the lack of compliance of some patients in recommended check-ups. 

Nevertheless, our study is an attempt to characterize this disease in our country, because (as many reports says) primary aldosteronism is not rare (not only when there is typical association hypertension - hypokalemia), but a more common pathology, which is necessary to take into account in the initial assessment of a new case of hypertension.
